# Optical skipping rope induced transverse OAM for particle orbital motion parallel to the optical axis

**DOI:** 10.1515/nanoph-2023-0551

**Published:** 2023-11-13

**Authors:** Liuhao Zhu, Xiaohe Zhang, Guanghao Rui, Jun He, Bing Gu, Qiwen Zhan

**Affiliations:** Advanced Photonics Center, School of Electronic Science & Engineering, Southeast University, Nanjing 210096, China; School of Automatic, Nanjing University of Information Science & Technology, Nanjing 210044, China; School of Physics and Electronics, Central South University, Changsha 410012, China; Collaborative Innovation Center of Light Manipulations and Applications, Shandong Normal University, Jinan 250358, China; School of Optical-Electrical and Computer Engineering, University of Shanghai for Science and Technology, Shanghai 200093, China

**Keywords:** physical optics, orbital angular momentum, optical tweezers

## Abstract

In structured light tweezers, it is a challenging technical issue to realize the complete circular motion of the trapped particles parallel to the optical axis. Herein, we propose and generate a novel optical skipping rope via combining beam shaping technology, Fourier shift theorem, and beam grafting technology. This optical skipping rope can induce the transverse orbital angular momentum (OAM) (i.e., nominal OAM, whose direction is perpendicular to the optical axis) and transfer it to the particles, so that the particles have a transverse torque, thereby causing the particles to rotate parallel to the optical axis. Experimentally, our optical tweezers validate that the designed optical skipping rope realizes the orbital motion of polystyrene particles parallel to the optical axis. Additionally, the experiments also demonstrate that the optical skipping ropes manipulate particles to move along the oblique coil trajectory and three-dimensional (3D) cycloidal trajectory. Using the laser beam induced OAM, this innovative technology increases the degree of freedom for manipulating particles, which is of great significance for the application of optical tweezers in optical manipulation, micromechanics, and mimicry of celestial orbits.

## 1 Introduction

Since the 1970s, the optical tweezers technology invented by Ashkin [1] has revolutionized a wide range of fields including condensed matter physics [2, 3], biology [4, 5], and nanomaterial science [6–8]. By exploiting the photomechanical properties of focused laser beams, optical tweezers technology enables various optical operations, such as fixation [1], movement [9], spin [10], rotation [11], levitation [12], sorting [13], and stretching of tiny particles [14]. At the same time, researchers have investigated various types of spatially structured beams, including Gaussian beams [15], vortex beams [16], vector beams [17], and vector vortex beams [18–20], to capture and manipulate particles. In brief, the spatially structured beams play an important role in the field of optical tweezers due to their rich wavefront distribution and high plasticity of the light intensity distribution.

As mentioned above, the optical field manipulation technology provides additional degrees of freedom for the beam and enriches the motion trajectories of the trapped particles in optical tweezers. The most typical example is the realization of the orbital motion of particles around the optical axis with vortex beams [21, 22]. Specifically, the vortex beam interacts with the particles to transfer the longitudinal orbital angular momentum (OAM) carried by the beam to the particles, so that the particles have a longitudinal torque, causing the particles to make orbital motion in the transverse plane perpendicular to the optical axis. Similarly, if the beam carrying the transverse OAM is used in optical tweezers, the particles will perform a circular motion parallel to the optical axis. Note that Zhan’s group has recently generated spatiotemporal optical vortices with controllable transverse OAM [23]. Due to its strict requirements for experimental equipment and environmental conditions, there is no report on particle manipulation using spatiotemporal optical vortices. Very recently, this transverse OAM has been employed for the self-rotation of nanorods with high frequency [24].

In the past decade, various three-dimensional (3D) beams have been proposed to try to realize the longitudinal circular motion of particles. Holographic optical tweezers are the first to realize 3D operation by adjusting the focusing position of the beam [25]. Due to its reliance on parametric equations and rotation matrices to manipulate the focus position, achieving parallel rotation to the optical axis in 3D space has proved to be challenging [15]. Another type of 3D particle manipulation is the tractor beam, which pushes and pulls particles [26, 27]. The tractor beam is good at realizing the round-trip motion of particles in the direction of light propagation, but it is still a challenge to realize the complete circular motion parallel to the optical axis [12]. A novel method for achieving 3D movement of particles involves the use of a recently developed shapeable structured beam [28–32], which allows the free customization of transport trajectories along the parametric equations. Despite the availability of alternative methods that do not rely on the parametric equations [31], the limitations of the shapeable structured beam still exist. Specifically, it cannot flip, so it can only produce OAM at a certain angle to the *z*-axis, and cannot generate the transverse OAM. Very recently, the concept of rigid-body optical tweezers is proposed, but this is not conducive to the independent control of multiple targets [33]. So far, the optical tweezers lack a straightforward, reliable, and user-friendly technology to achieve the complete circular motion of particles parallel to the optical axis.

In this work, we propose and generate a novel optical skipping rope by synergistically combining the 3D circular trajectory equation with both the beam grafting technique and the Fourier phase shifting technique. The optical skipping rope allows for a selective and adjustable generation of capture points and enables 360° flipping. Intriguingly, laser beam induced transverse OAM increases the degree of freedom for manipulating particles, which can realize the circular motion of particles parallel to the optical axis. Experimentally, our optical tweezers demonstrate that the designed optical skipping ropes manipulate polystyrene particles to move along circular motion parallel to the optical axis, oblique coil motion, and 3D cycloidal motion.

## 2 Methods

**Figure 1:**
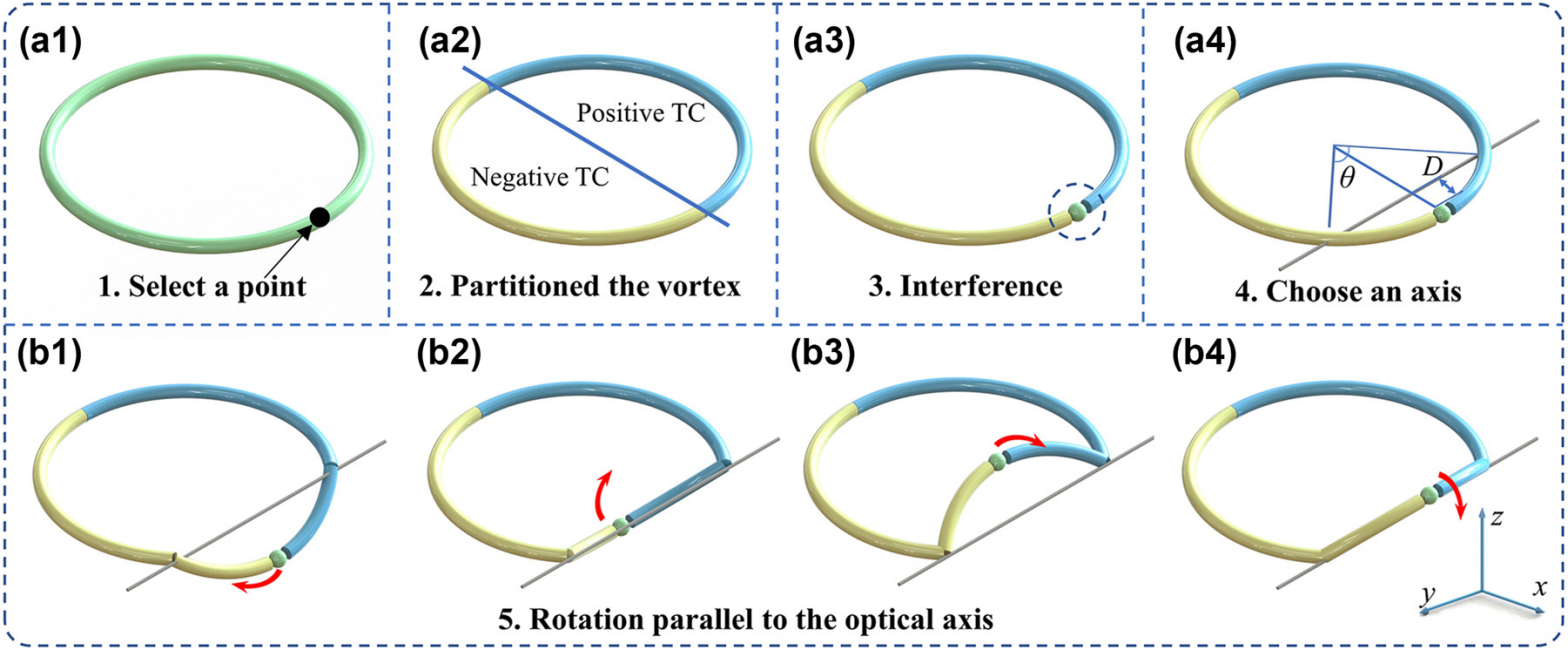
The generation principle of optical skipping rope. (a1) the manipulation point needs to be selected, which determines the position of the captured particles, (a2) the vortex beam is divided into two parts with opposite TC, (a3) the interference superposition is performed at the capture point, which generates the capture point, (a4) the rotation axis is selected, (b1)–(b4) a portion of the beam is rotated along the rotation axis to drive the particles for longitudinal movement.

To generate the optical skipping rope, firstly, one needs a 3D shapeable beam. In the initial plane, the computer-generated hologram of the structured optical field with an arbitrary curvilinear mode is represented as [28]

(1) $$\begin{array}{ll} G\left(x,y,\tau \right) & =\frac{1}{\overset{T}{\underset{0}{\int }}\left|{c^{\prime }}_{0}\left(\tau \right)\right|\text{d}\tau }\overset{T}{\underset{0}{\int }}\\ & \;\times exp\left\{j\pi \frac{{\left[x-x_{0}\left(\tau \right)\right]}^{2}+{\left[y-y_{0}\left(\tau \right)\right]}^{2}}{\lambda f^{2}}z_{0}\left(\tau \right)\right\}\\ & \;\times \psi_{0}\left(x,y,\tau \right)\left|{c^{\prime }}_{0}\left(\tau \right)\right|\text{d}\tau , \end{array}$$

where *λ* is the wavelength of light and *f* is the focal length of the lens. *x*_0_(*τ*), *y*_0_(*τ*), and *z*_0_(*τ*) are the parametric equation of the curve, |*c*′_0_(*τ*)| = [*x*′_0_(*τ*)^2^ + *y*′_0_(*τ*)^2^ + *z*′_0_(*τ*)^2^]^1/2^ dominates the shape of the beam with *τ*∈ [0, *T* = 2π], *c*′_0_(*τ*) denotes *c*_0_(*τ*) taking the derivative of *τ*, *ψ*_0_(*x*, *y*, *τ*) represents the phase of the beam. The presented work mainly takes circles as an example. In this case, the parametric equation of the curve is written as

(2) $$\left\{\begin{array}{cc} x_{0}\left(\tau \right) & =R_{0}\cos\tau \\ y_{0}\left(\tau \right) & =R_{0}\sin\tau \\ z_{0}\left(\tau \right) & =0 \end{array}\right.,$$

where *R*_0_ is the radius of the vortex beam. Note that this beam is produced by a loop integral along a predesigned curve. Therefore, it is possible to conveniently cut and graft the optical field by controlling the integration process. Next one needs to select a control point on the annular beam shown in Figure 1(a1), the vortex beam is divided into two sections via the diameter of this point. These two segments each have topological charges (TCs) with opposite signs. To make the capture easier, we choose to extend two semi-circular beams at the capture point. In this way, due to TCs with opposite signs on two semicircles, the interference will occur at the capture point to produce an interference point with strong gradient force, as shown in Figure 1(a3). Then a chord is chosen as the rotation axis for the capture point. At the same time, the position of the rotation axis is controlled by adjusting the circumference angle corresponding to the chord. Naturally, the height of the arch *D* becomes the radius of rotation of the captured particle parallel to the optical axis. Then, the arch corresponding to the axis of rotation in 3D space is synchronously rotated by calculating the parametric equations for 3D circles. Due to the arch’s rotation, the trapping point will inevitably induce the transverse OAM. It is noteworthy that the transverse OAM induced by this optical skipping rope is a nominal OAM with its direction perpendicular to the optical axis. This OAM vector is directly determined by the rotational velocity of the captured point, which can be expressed by the formula **L** = ***r*** × ***P***. Here ***r*** denotes the particle’s position vector relative to an origin. ***P*** = *m* ***v*** represents the particle’s momentum, where ***v*** is the rotational speed and *m* is the mass of the particle. As illustrated in Figure 1, only part of the beam needs to be rotated, so the components that need to be rotated do not use the same formula as the rest of the components. This implies that in the region of the beam depicted in Figure 1 that has undergone rotation, there exists a normal vector, denoted as ***n*** (*n*_*x*_, *n*_*y*_, *n*_*z*_), that is a variable, while the non-rotated segment retains its original normal vector ***n***_0_ (0, 0, 1). It is assumed that the normal vector is ***n*** (*n*_*x*_, *n*_*y*_, *n*_*z*_+Δ*n*) (Δ*n* is an additional constant to prevent the rotation from separating), where the angle of rotation *φ* determines *n*_*z*_ and the relationship between them is *n*_*z*_ = tan*φ*. It is worth noting that *φ* is a quantity that varies uniformly over time, and if the time factor is *t*, then *φ* = *t*Δ*φ* (Δ*φ* is a parameter that determines rotation speed). Accordingly, the transverse OAM induced by the generated optical skipping rope can be expressed as

(3) $$L_{t}=mD^{2}\Delta \varphi .$$

This means that for a fixed particle, the larger the height of the arch *D*, the larger the induced transverse OAM; while for a certain optical skipping rope, the greater the particle’s mass, the larger the induced transverse OAM.

The parametric equations of a space circle can be calculated via normal vectors

(4) $$\left\{\begin{array}{c} x_{0}\left(\tau \right)=R_{0}\left(a_{x}\cos\tau +b_{x}\sin\tau \right)\\ y_{0}\left(\tau \right)=R_{0}\left(a_{y}\cos\tau +b_{y}\sin\tau \right)\\ z_{0}\left(\tau \right)=R_{0}\left(a_{z}\cos\tau +b_{z}\sin\tau \right) \end{array}\right.,$$

where ***a*** = (*a*_*x*_, *a*_*y*_, *a*_*z*_) = ***n*** × (1, 0, 0), ***b*** = (*b*_*x*_, *b*_*y*_, *b*_*z*_) = ***n × a***. Because of the change in the spatial capture position, it is also necessary to use Fourier shift theorem to prevent it from separating during rotation. In the case, the computer-generated hologram of the optical skipping rope with *k* parts is represented as

(5) $$\begin{array}{ll} G_{0}\left(x,y,\tau_{0}\right) & =U_{1}G_{1}\left(x,y,\tau_{1}\right)+U_{2}G_{2}\left(x,y,\tau_{2}\right)\\ & \;+U_{3}G_{3}\left(x,y,\tau_{3}\right)+\cdots \cdots +U_{k}G_{k}\left(x,y,\tau_{k}\right), \end{array}$$

here *U*_*k*_ is a Fourier shift factor expressed as *U*_*k*_ = exp[*j*2π(*ξ*_*k*_ *x* + *η*_*k*_ *y*)], where the values of *ξ*_*k*_ and *η*_*k*_ are related to the parameters of the experimental device, which should be set in combination with the experimental conditions. Specifically, we take *ξ*_*k*_ = 256 and *η*_*k*_ = 490 in our experiment. When the result in Figure 1 is desired, one chooses *k* = 4, *τ*_1_ ∈ [0, π − *θ*/2], *τ*_2_ ∈ [π − *θ*/2, π + *σ*], *τ*_3_ ∈ [π − *σ*, π + *θ*/2], and *τ*_4_ ∈ [π + *θ*/2, 2π]. The calculation shows that one needs to set *σ* = π/*l* to obtain perfectly interference with the capture point [32], where *l* = 30 is the TC value of the optical skipping rope. Hence, the optical skipping rope can be achieved with the help of Fourier transform.

## 3 Experimental setup

**Figure 2:**
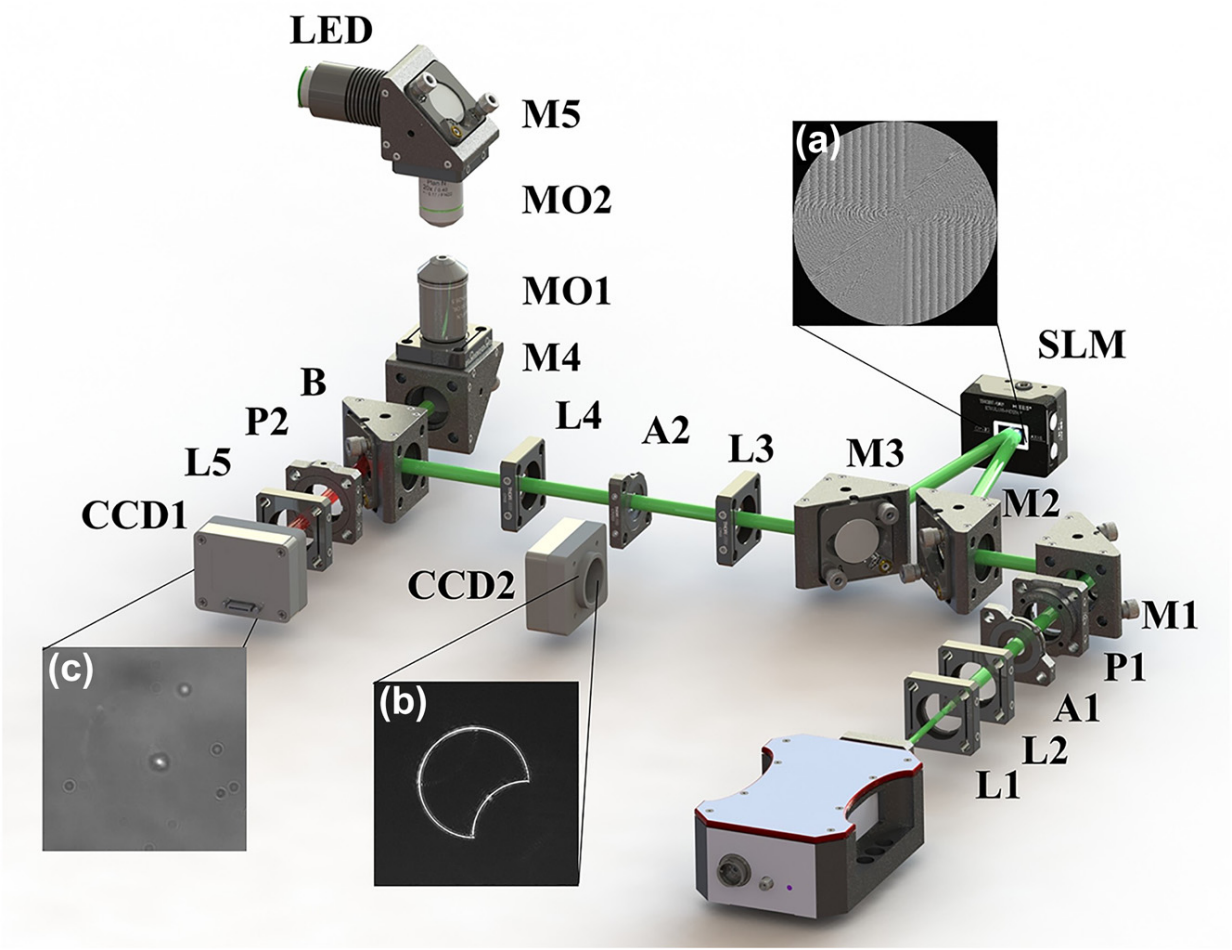
Schematic of the experimental setup. L1, concave lens; L2–L5, convex lenses; P1 and P2, polarizers; A1 and A2, apertures; SLM, spatial light modulator; M1–M5, mirrors; CCD1 and CCD2, charge-coupled devices; MO1 and MO2, microscopic objectives; LED, backlighting source; B, band-pass filter. (a) Phase mask diagram, (b) beam intensity before tightly focusing, and (c) particle manipulation diagram.

**Figure 3:**
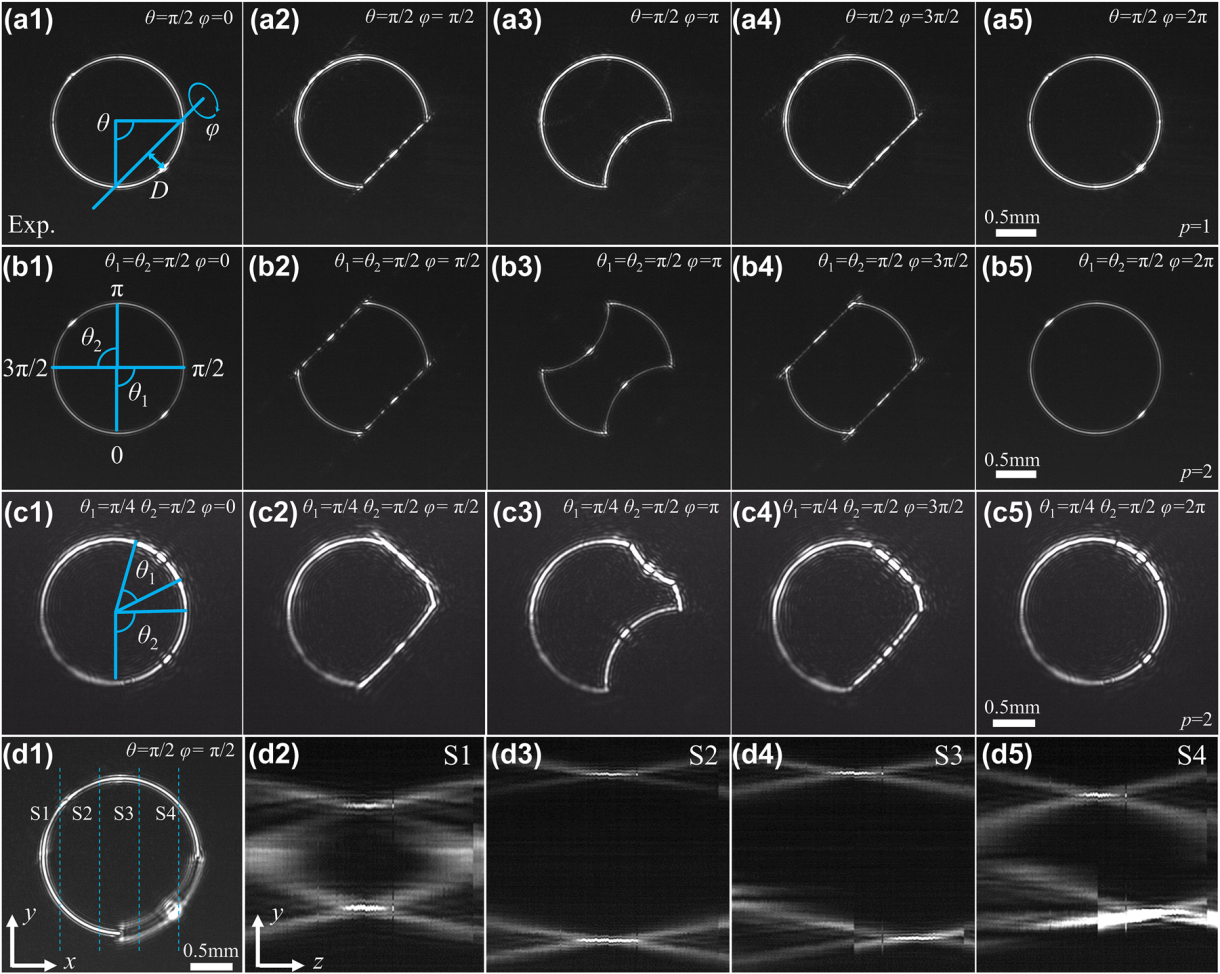
Experimentally measured intensity distributions of optical skipping ropes with different parameters of *θ*, *φ*, and *p*. (a1)–(a5) intensity distributions of the optical skipping ropes with *θ* = π/2 and different *φ*, for the single-particle manipulation (*p* = 1). (b1)–(b5) Intensity distributions of the optical skipping ropes with *θ*_1_ = *θ*_2_ = π/2 and different *φ*, for the symmetric two-particle manipulation (*p* = 2). (c1)–(c5) Intensity distributions of the optical skipping ropes with *θ*_1_ = π/4, *θ*_2_ = π/2, and different *φ*, for the asymmetric two-particle manipulation (*p* = 2). (d1) 2D intensity distribution of an optical skipping rope with *θ* = π/2 and *φ* = π/4. (d2)–(d5) Measured intensity patterns at planes (S1 to S4) with separation of 0.5 mm marked in (d1).

To generate the optical skipping rope proposed above and subsequently manipulate particles, Figure 2 shows an experimental setup of optical tweezers including the holographic technology. The laser beam with a wavelength of *λ* = 532 nm is produced by a continuous-wave laser with a power adjustable from 0 to 4 W. The output Gaussian beam is expanded and collimated by the combination of a concave lens (L1, *f* = −5 cm) and a convex lens (L2, *f* = 15 cm) to form a parallel beam, and the uniform illumination part is taken as a flat-top beam with the apertures (A1). The flat-top beam becomes a linearly polarized light after passing through a polarizer (P1), then it is reflected by the mirrors (M1, M2) and incidents to the spatial light modulator (SLM) at an angle of less than 5°. The SLM is loaded with a predesigned phase mask. According to the generation principle of phase holographic plates, it is essential to initially perform plane wave phase interference on the phase of the target beam. Subsequently, to improve the beam quality, it is necessary to add an annular diaphragm. Therefore, the phase mask can be expressed as *T* (*x*, *y*) = circ [(*x* ^2^ + *y* ^2^)^1/2^] × exp{*j*·angle[*G*_0_(*x*, *y*, *τ*_0_)] + *j*2π*x*/*d*}, where angle[ ] is a function of the phase angle and *d* is the period of the blazed grating. The beam modulated by the above phase mask passes through the mirror (M3) and enters the coupled optical path composed of a 4*f* system (L3, *f* = 20 cm; L4, *f* = 20 cm). Meanwhile, the aperture (A2) only allows +1 order diffracted light to pass through. B is a band-pass filter with the central wavelength of 620 ± 6 nm. The coupled optical path mainly controls that the optical skipping rope can perfectly reflect into the microscopic objective (MO1, oil, 100×, NA = 1.49), and then be tightly focused by the microscopic objective to manipulate particles. The background light source is an LED with the wavelength of 620 ± 10 nm, which is focused by a microscopic objective (MO2, 10×, NA = 0.3) and recorded by the camera (CCD1, 1280 × 1024 pixels, pixel size: 5.3 × 5.3 μm^2^). Note that when it is necessary to photograph the intensity distribution of the beam before tightly focusing, CCD2 should be placed at A2 position. The experimental results in Figure 3 are all captured at this position.

## 4 Results and discussion

The adjustability of spatially structured beam directly determines its applicability. Therefore, it is necessary to modulate the optical skipping rope, verify the influence of each parameter on the optical skipping rope, and analyze its possible role in particle manipulation applications. First, let’s look at the manipulation of individual particle in more detail and set the number of manipulated particles to *p* = 1. Here, setting *θ* = π/2, Figure 3(a) shows the measured intensity distributions of optical skipping ropes with different rotation angles *φ*. In manipulation applications, it is preferred to change the height of an arch *D* by setting the angle *θ*. Next, one needs to give the conversion relationship between the angle *θ* and the height *D* by *D* = *R*_0_[1 − cos(*θ*/2)] (Unless otherwise specified, all experiments in this work are taken as *R*_0_ = 0.6 mm). The optical skipping ropes for single-particle manipulation are shown in Figure 3(a1)–(a5). It is seen that a part of the optical skipping rope rotates parallel to the optical axis along the rotation axis (For more details, see Visualization 1, which shows the full 360° rotation). To gain an intuitive understanding of the generated light field, it is imperative to experimentally measure the 3D intensity distribution of representative optical skipping ropes. Figure 3(d1) shows the measured intensity distributions in the *x*–*y* plane for an optical skipping rope with *θ* = π/2 and *φ* = π/4, while Figure 3(d2)–(d5) are the measured intensity patterns at planes from S1 to S4 with the separation of 0.5 mm marked in (d1). As shown in Figure 3(d2)–(d5), it is found that the top and bottom focal positions at S1 and S2 planes are identical, whereas the focal positions at S3 and S4 planes move to the right, indicating that a portion of the beam has already rotated. Then, we select the corresponding two points for the symmetric two-particle manipulation. When simultaneous control of two or more control points is required, the introduction of a new position parameter, denoted as *q*_*i*_, becomes imperative. As depicted in Figure 3(b1), the entire circular domain can be likened to a clock, encompassing a range of 0–2π. In this scenario, selecting *q*_1_ = π/4 and *q*_2_ = 5π/4. The parameters should take as *k* = 6, *θ*_1_ = *θ*_2_ = π/2, *τ*_1_ ∈ [0 − *σ*, *θ*_2_/2], *τ*_2_ ∈ [*θ*_2_/2, π − *θ*_1_/2], *τ*_3_ ∈ [π − *θ*_1_/2, π + *σ*], *τ*_4_ ∈ [π + *σ*, π + *θ*_1_/2], *τ*_5_ ∈ [π + *θ*_1_/2, 2π − *θ*_2_/2], *τ*_6_ ∈ [2π − *θ*_2_/2, 2π + *σ*], where *θ*_1_ and *θ*_2_ are the parameters controlling the arch height of two different capture points, respectively. Accordingly, the experimentally measured intensity distributions are shown in Figure 3(b1)–(b5) (For more details, see Visualization 2, which shows the full 360° rotation). And finally, two asymmetric captured point is obtained via setting the parameter *q*_1_ = π/4, *q*_2_ = 3π/4, *k* = 6, *θ*_1_ = π/4, *θ*_2_ = π/2, *τ*_1_ ∈ [0 − *σ*, *θ*_2_/2], *τ*_2_ ∈ [*θ*_2_/2, π/2 − *θ*_1_/2], *τ*_3_ ∈ [π/2 − *θ*_1_/2, π/2 + *σ*], *τ*_4_ ∈ [π/2 − *σ*, π/2 + *θ*_1_/2], *τ*_5_ ∈ [π/2 + *θ*_1_/2, 2π − *θ*_2_/2], *τ*_6_ ∈ [2π − *θ*_2_/2, 2π + *σ*]. Figure 3(c1)–(c5) show the measured intensity distributions of the optical skipping ropes with *φ* = 0 − 2π, for asymmetric two-particle manipulation (*p* = 2) (For more details, see Visualization 3, which shows the full 360° rotation).

**Figure 4:**
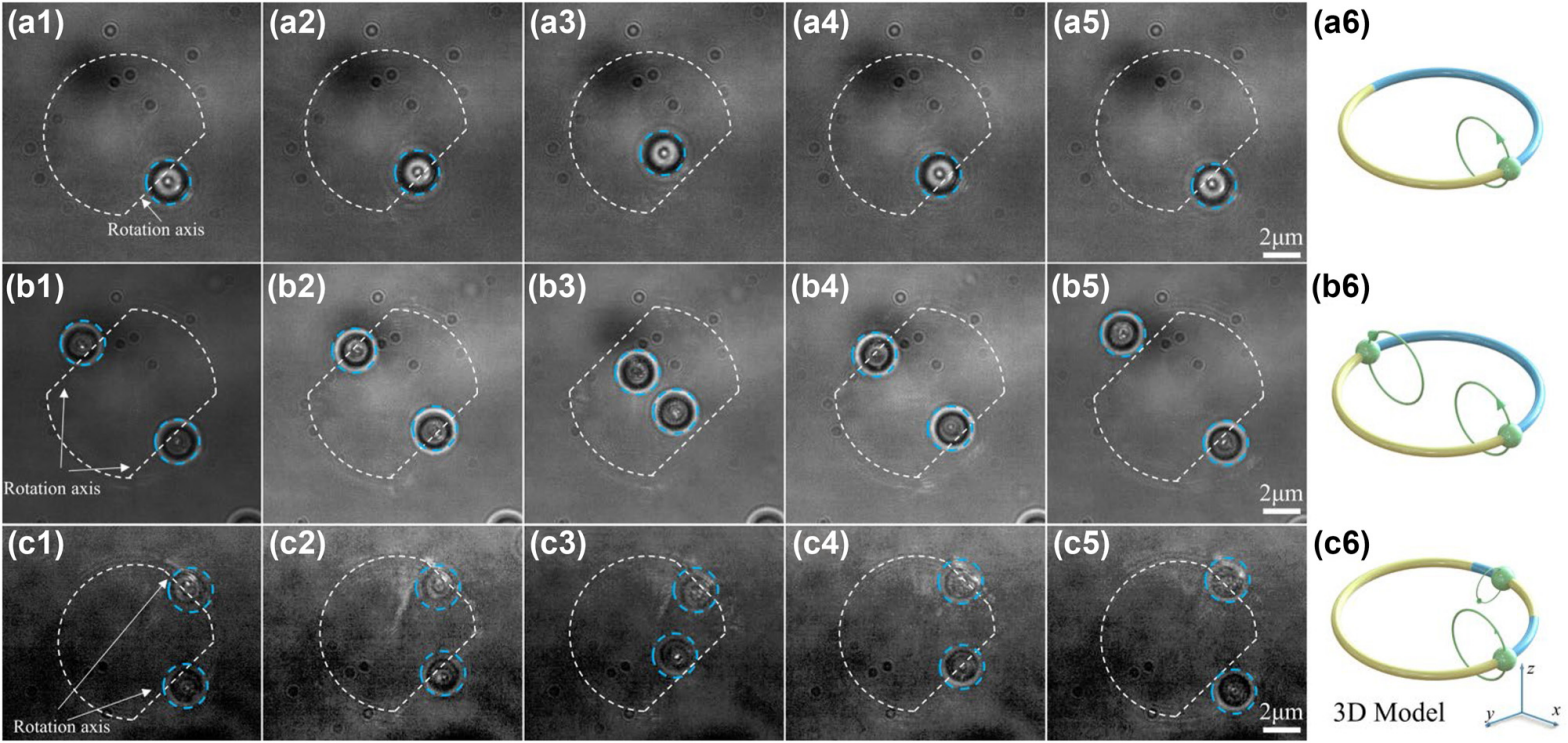
Optical skipping ropes manipulating the particle to rotate parallel to the optical axis. (a1)–(a5) Single-particle rotation, (b1)–(b5) symmetric two-particle rotation, and (c1)–(c5) asymmetric two-particle rotation. The white dashed lines represent the outline of the beam and its axis of rotation. (a6), (b6), and (c6) are their corresponding 3D model, respectively.

To demonstrate the ability of optical skipping ropes to induce particle rotation parallel to the optical axis, experimental verification is required. In our experiment, polystyrene pellets were employed as the object particles of manipulation. Firstly, we used the optical skipping rope illustrated in Figure 3(a1)–(a5) to operate polystyrene pellets with a diameter of *R*_*p*_ = 3 μm, and the outcomes of the particle manipulation are depicted in Figure 4(a1)–(a5) (For additional details, refer to Visualization 4. The change rate Δ*φ* in Visualization 4 is π/50 per second). Additionally, Figure 4(b1)–(b5) present a diagram of two particles being manipulated by the optical skipping ropes in Figure 3(b1)–(b5) (For more details, see Visualization 5. The change rate Δ*φ* in Visualization 5 is π/50 per second). Finally, the results of asymmetrical manipulation of two particles by the optical skipping ropes in Figure 3(c1)–(c5) are shown in Figure 4(c1)–(c5) (For more details, see Visualization 6. The change rate Δ*φ* in Visualization 6 is π/50 per second). From the manipulation video, it is evident that the particles successfully move in a complete circle parallel to the optical axis under the irradiation of the optical skipping rope. This is because the rotation of the beam causes the particles to constantly deviate from the center of the capture point, resulting in a gradient force, the direction of which is always tangent to the circumference of the rotating optical skipping rope. The principle of particle rotation induced by this method is different from that caused by direct optical radiation force, although the effect of particle rotation is similar. The underlying mechanism of the experimental observations is explained as follows: the optical skipping rope induces the transverse OAM described by Eq. (3) and transfers it to the particles, which makes the particles have a transverse torque, causing the particles to rotate parallel to the optical axis. Without a doubt, the realization of such particle motion provides the possibility for colloidal assembly in the microscopic world, and even for the construction of an assembler using spatially structured light.

To optimize the practicality of the presented technology, we analyze the maximum velocities in various scenarios to manipulate the movement of particles with different sizes. Our approach involves a test that affects the circular motion of polystyrene particles. Specifically, with the increasing of the Δ*φ* value, the critical particle’s velocity is recorded when the particles could no longer complete a circle. This velocity represents the maximum velocity at which the optical skipping rope can support particles to complete circular motion. In this study, two comparative measurement experiments are conducted (The laser power is kept at 600 mW). In the first measurement experiment, the diameter of the particle is *R*_*p*_ = 1 μm, and the beams’ radii are set to *R*_0_ = 0.5 mm, 0.6 mm, and 0.7 mm, respectively. Correspondingly, for the optical skipping ropes with *θ* = π/2, the maximum velocities of particles are measured to be *v*_max_ = 2.36 μm/s, 1.41 μm/s, and 0.33 μm/s, their transverse OAMs are calculated to be *L*_*t*_ = 9.90 × 10^−11^ g μm^2^/s, 7.13 × 10^−11^ g μm^2^/s, and 1.94 × 10^−11^ g μm^2^/s, respectively. As theoretically expected, we observe that the maximum velocity of particles decreases as the beam radius increases. This is because the larger the radius of particle rotation, the greater the centripetal force required. Therefore, at a constant light intensity, the maximum velocity it can support becomes slower and slower. This phenomenon can be attributed to the increase of torque and centripetal force required to maintain the orbital motion. In the second experiment, we focus on particles with a diameter of *R*_*p*_ = 2 μm and a beam’s radius of *R*_0_ = 0.6 mm with *θ* = π/2. The maximum velocity that can support particles to make orbital motion is measured to be *v*_max_ = 0.85 μm/s. Combined with the first experimental results of *R*_*p*_ = 1 μm, *R*_0_ = 0.6 mm, *θ* = π/2, and *v*_max_ = 1.41 μm/s, it is shown that the larger the particle size, the slower the motion speed. This is because larger particle experience greater viscous resistance, leading to a decrease in their velocity. In short, we generated the optical skipping rope and demonstrated its function of manipulating particles to make the orbital motion parallel to the optical axis. This work only provides a glimpse of the potential for optical skipping ropes, as the partitioning of the ring presented in this study is arbitrary. In practical applications, users can easily select combination points based on specific situational requirements. The next step is to extend our technique with two usage examples.

**Figure 5:**
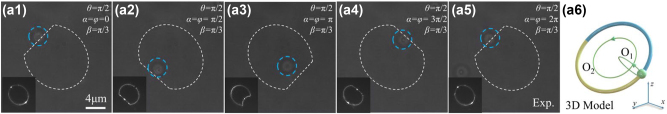
Optical skipping ropes manipulating particles to move along an oblique coil trajectory. (a1)–(a5) experimental screenshot, the white dashed lines represent the outline of the beam and its axis of rotation. The lower left inset of each picture is the intensity distribution of the corresponding optical skipping rope. (a6) 3D Model of the optical skipping rope and schematic of orbit. O1 is the motion orbit of the particle, O2 is the motion orbit of the center of O1, and the arrow shows the direction.

In the aforementioned scenario, we employ a stationary capturing arch that is oriented parallel to the *x*–*y* plane. It is noteworthy that the said arch can also be dynamically adjusted to achieve greater flexibility in controlling the trajectory of particle according to specific requirements. For example, according to the configuration depicted in Figure 4, it is now necessary to implement the particle manipulation illustrated in Figure 5(a6). Consequently, the normal vector of the stationary segment of the circular equation should be modified as ***n***_0_ (0, tan*β*, 1). This means that the entire optical skipping rope has an angle of π − *β* with respect to the *x*–*y* plane. Figure 5 shows the experimental results of optical skipping ropes manipulating particles to move along an oblique coil trajectory when *β* = 60°. To achieve the outcome shown in Figure 5, we introduce an initial parameter value *α* and set the parameters of *k* = 4, *τ*_1_ ∈ [0 + *α*, π − *θ*/2 + *α*], *τ*_2_ ∈ [π − *θ*/2 + *α*, π + *σ* + *α*], *τ*_3_ ∈ [π − σ + *α*, π + *θ*/2 + *α*], and *τ*_4_ ∈ [π + *θ*/2 + *α*, 2π + *α*]. This rotational adjustment causes orbit O_1_ to rotate along the trajectory of orbit O_2_. It is pertinent to observe that *α* is a variable quantity, and the motion trajectory of particle is determined by its relationship with *φ*. In the case illustrated in Figure 5, we take *α* = *φ*. Therefore, as the particle completes one revolution around the *z*-axis, it precisely returns to its initial position by following the trajectory of orbit O_2_ (For additional details, refer to Visualization 7. The change rate Δ*φ* in Visualization 7 is π/100 per second). Similar to *φ*, the parameters of *α* and *β* are also time-dependent, where *α* = *t*Δ*α* and *β* = *t*Δ*β*. The orbital radius and rotation speed of particles can be independently adjusted by controlling the parameters *φ*, *α*, and *β*. In other words, OAM induced by optical skipping rope is no longer limited to transverse OAM. Since the inclination angle of the three orbits and the changing velocity are freely controlled, i.e., the induced OAM can be controlled in any direction at this time. The velocity vectors generated by orbits *φ*, *α*, and *β* are assumed to be ***v***_1_, ***v***_2_, and ***v***_3_, respectively. Hence, the induced OAM vector can be expressed as

(6) $$L=mD\left(v_{1}\times v_{2}\times v_{3}\right).$$

Here, the three velocities follow the principle of vector superposition, and the induced OAM of their superposition can be oriented in any 3D direction. By manipulating only three variables, namely, *φ*, *α*, and *β*, a helical particle trajectory can be achieved, which can incline at any desired angle with any number of turns. Compared with other methods such as holographic optical tweezers [15] and shaped beams [34, 35] that involve using inverse integration of diffraction theory to calculate motion paths, our approach is significantly simpler and conserves computational resources.

**Figure 6:**
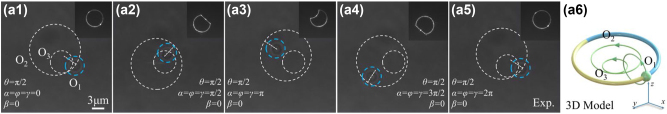
Optical skipping ropes manipulate particles to move along a 3D cycloidal trajectory. (a1)–(a5) experimental screenshot, the white dotted line is orbit O1, the larger virtual circle is orbit O2, and the smaller dotted line is O3. The upper right inset of each picture is the intensity distribution of the corresponding optical skipping rope. (a6) 3D model of the optical skipping rope and schematic of orbit. O1 is the motion orbit of the particle, O2 is the motion orbit of the center of O1, O3 is the motion orbit of the center of O2, and the arrow shows the direction.

Undoubtedly, the potential of optical skipping ropes extends well beyond the aforementioned applications. For instance, by setting *β* = 0 and *α* = *φ*, both the particle and the center of orbit O_1_ would rotate around orbit O_2_, as depicted in Figure 6(a6). Furthermore, we introduce a rotation parameter for the entire optical skipping rope *U*_0_, which is expressed as follows: *U*_0_ = exp[*j*2π(*ξ*_0_ *x*cos*γ* + *η*_0_ *y*sin*γ*)], where *γ* is the parameter that controls the rotation speed in the orbit O_3_. When *γ* = *φ*, the center of orbit O_1_ will rotate one around orbit O_2_ while the center of orbit O_2_ will rotate one around orbit O_3_. And the total computer-generated hologram of this beam is expressed as

(7) $$\begin{array}{ll} G_{0}\left(x,y,t_{0}\right) & =U_{0}z\left[U_{1}G\left(x,y,\gamma_{1}\right)+U_{2}G_{2}\left(x,y,\gamma_{2}\right)\right.\\ & \;+\left.U_{3}G_{3}\left(x,y,\gamma_{3}\right)+\cdots \cdots +U_{k}G_{k}\left(x,y,\gamma_{k}\right)\right]. \end{array}$$

As a result, we obtain a trajectory comprising of three coexisting orbits, as illustrated in Figure 6(a6) (For additional details, refer to Visualization 8. The change rate Δ*φ* in Visualization 8 is π/100 per second). The actual trajectory of a particle in 3D space corresponds to a 3D helical cycloid. Significantly, the parameters *α*, *β*, and *γ* are independently adjustable, thereby conferring independent degrees of freedom to orbits O_1_, O_2_, and O_3_. It should be emphasized that although we have demonstrated the longitudinal manipulation of a single particle, the optical skipping ropes can also capture and manipulate multiple particles simultaneously, such as the two-particle manipulation shown in Figure 4. In this sense, it can mimic the trajectory of celestial bodies at a microscopic scale, such as the planet rotates around the star. Although the sample capture points described in this work are symmetric, it is noteworthy that the selection of capture points does not have to be symmetric (e.g., see Figure 4(c)). Furthermore, the number of capture points is not limited to one or two; it can be any number of multiple capture points as you desire. This technology is scalable, and future enhancements can be achieved by integrating optical skipping rope with other curves [31, 36], thereby enabling a broader range of particle-capturing methods.

## 5 Conclusions

In summary, we have combined the 3D circular trajectory equations with both beam grafting technique and Fourier phase shifting technique to propose, design, and generate a novel structured light beam, named optical skipping rope. This optical skipping rope allows for a selective and adjustable generation of capture points and enables 360° flipping. Intriguingly, the designed optical skipping rope induces a transverse OAM, which makes the particles have a transverse torque, thereby causing the particles to rotate parallel to the optical axis. Our optical tweezers experiments have validated that the generated optical skipping rope drives the orbital motion of polystyrene particles parallel to the optical axis. Additionally, we have experimentally demonstrated the effectiveness of the optical skipping rope in particle trajectory design by creating two trajectories, namely an oblique coil trajectory and a 3D cycloidal trajectory. The proposed technique solves the longstanding problem that spatially structured light tweezers cannot manipulate particles to orbital motion parallel to the optical axis. The optical skipping rope is easy to implement, operate, and use, which injects new vitality into the application of structured light tweezers in optical manipulation, micromechanics, and mimicry of celestial orbits.
